# Helium-free MRI systems: What does that mean for a radiology decision maker?

**DOI:** 10.1186/s13244-026-02410-5

**Published:** 2026-09-29

**Authors:** Michael Friebe

**Affiliations:** 1 Otto-von-Guericke-University, Faculty of Medicine, Magdeburg, Germany; 2 AGH University of Krakow, Faculty of Computer Science, Krakow, Poland

**Keywords:** Superconductivity, Magnetic resonance imaging, Helium, Magnets

## Abstract

**Abstract:**

Traditional MRI systems rely on large liquid-helium baths to maintain superconductivity, requiring complex infrastructure, quench pipes, and ongoing helium supply management. Modern “dry” or sealed micro-helium MRI magnets replace this approach using conduction cooling and sealed helium volumes of only a few liters. These systems drastically reduce helium dependence, eliminate routine refilling, simplify installation, and lower the lifetime operating costs. The major practical advance came from moving from the open helium baths in the 2010s, which still required quench pipes and other safety features, to sealed systems, rather than from differences in small helium volumes (e.g., 0.7 vs. 7 L or 20 L) marketed by vendors. Smaller volumes marginally affect safety margins and resilience during power interruptions, rather than affecting routine clinical operations. This paper provides a brief introduction to the problem and terminology, and subsequently presents the technological issues related to the volume of sealed micro-helium MRI magnets. It also provides a list of questions to ask about the magnet sub-assembly itself, which enables the calculation of the total cost of ownership.

**Key Points:**

- **Question** What does it mean and how relevant is a “helium-free” MRI magnet and the remaining sealed liquid Helium volume?
- **Findings** The real operational leap in MRI magnet technology was from hundreds/thousands of liters of liquid helium to single-digit sealed systems; the sub-liter distinction does not really matter.
- **Clinical relevance statement** The article argues from a technical and economic perspective. While these sealed systems eliminate helium logistics, come with simpler siting, and reduced infrastructure costs, they raise other procurement questions for suppliers, like quench recovery time and resilience in low-resource settings.

**Graphical Abstract:**

**Figure:**
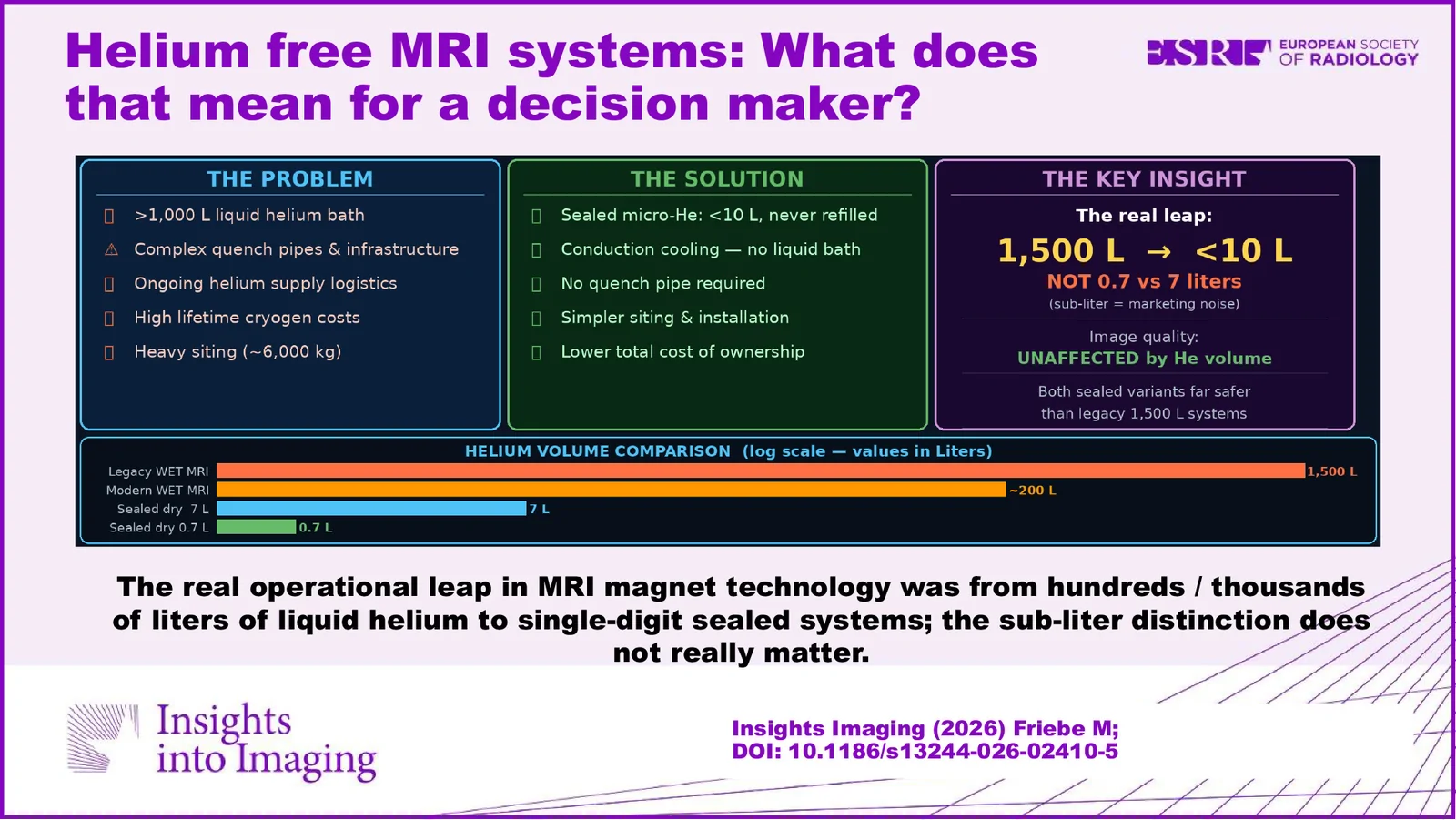

## Introduction to helium-free MRI magnets

Walking the industry exhibition of the 2025 RSNA in Chicago and most recently the 2026 ECR in Vienna, so-called “Helium-Free” superconducting magnets were presented as an important part of new MRI system offerings [1–3].

And yes, they do have advantages, but as always, you often buy advantages in one area with disadvantages somewhere else, most likely higher investment versus higher operational costs, as well as building and installation-related issues. The goal of this short statement paper is to explain the technical advancement, highlight the differences and clarify and demystify some of the claims.

**Fig. 1:**
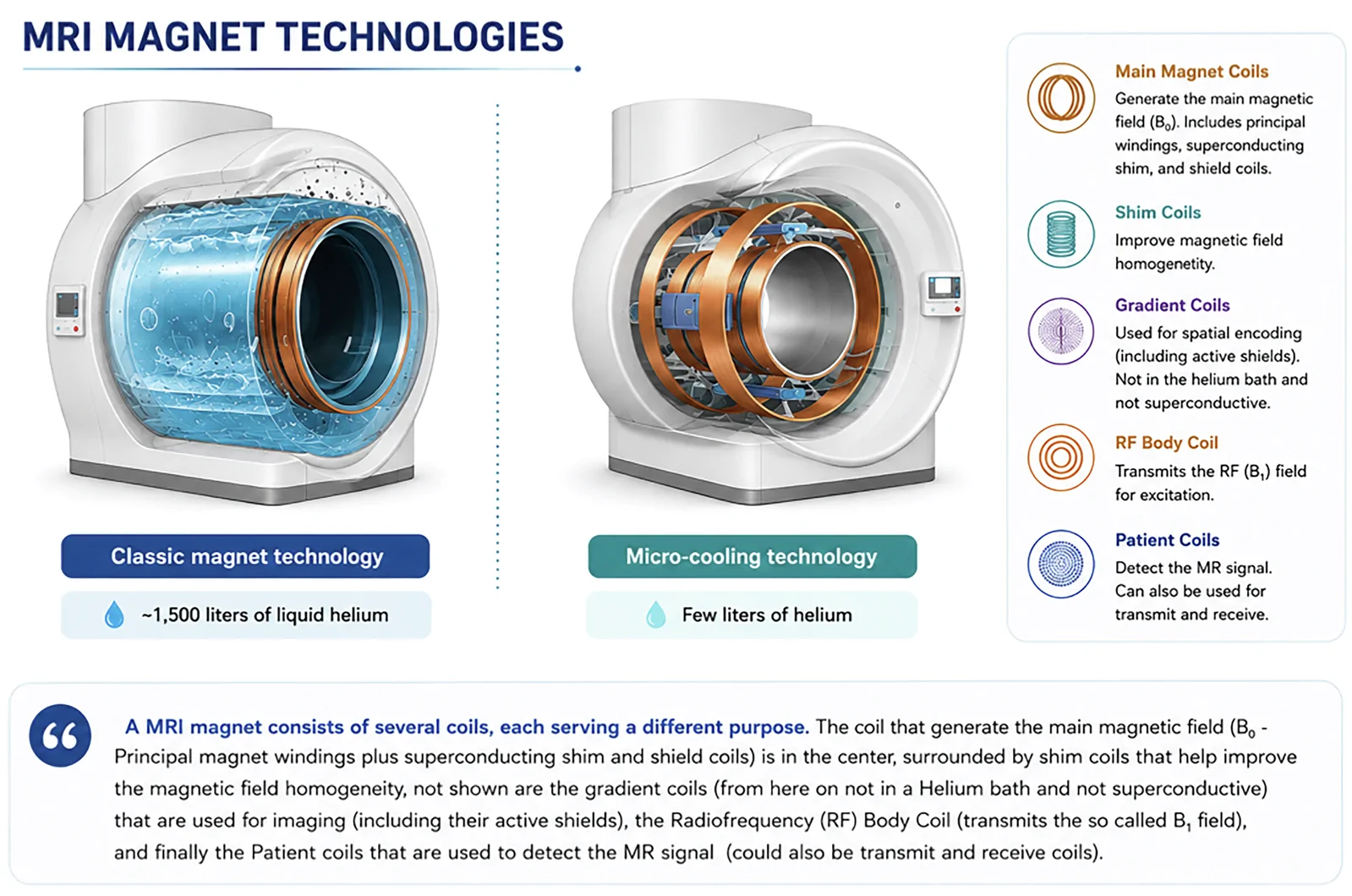
A classic MRI magnet on the left (around 1500 L of liquid Helium) and the new sealed micro-helium and conduction-cooled magnet (less than 10 L of liquid Helium). The reduced safety requirements lead to a greatly reduced weight (down from 6000 kg to about 2500 kg) and eliminate the need for a quench pipe that would guide the evaporated Helium gas to the outside in case of a magnet quench (loss of superconductivity)

In the traditional MRI world, the imaging magnet (typically 1.5 Tesla or 3 Tesla equivalent to approx. 30,000 to 60,000 times the earths magnet field) is basically a huge metal donut taking an ice bath in a swimming pool of liquid helium (the cryostat, with a temperature of around 4 K or −269 degrees C—the most often used material for the MRI coils, niobium-titanium [NbTi], becomes superconductive below 9.4 K), typically well over a thousand liters, just to keep the superconducting coils cold enough to behave (see Fig. 1 left).

Behavior here means being in a zero-electrical-resistance, superconducting state that allows a current in a coil to create a magnetic field inside the coil without requiring any electrical power to maintain the magnetic field. Continuous electrical energy is still needed, however, to cool the cryostat and maintain the liquid state at 4 K.

This “wet” design is wonderfully stable, but commits you to a lifelong relationship with helium suppliers, boil-off management (loss of liquid helium volume over time—typically in the order of 1–2 L per day despite cooling), and an infrastructure that assumes, at some point, all that helium might decide to escape at once in a dramatic quench. This can happen when the temperature of the cryostat gets high enough, causing a transition—due to the resistive heat of the current on the coil after losing superconductivity—from the liquid state to gas that causes an expansion of 750 times its original liquid volume at standard conditions (room temperature and normal atmospheric pressure), which comes with a huge pressure increase inside the magnet vessel. Without a pressure relief, this could lead to a very dangerous explosion.

That’s why most MRI systems need a pressure valve and a quench pipe to vent the gas safely out of the building, heavy structural support for several tons of magnet and cryostat, and a fair amount of safety engineering just so you can get nice brain pictures while nobody suffocates in the scan room [4].

The so-called helium-free or dry magnets flip this model by using conduction cooling with cryocoolers attached directly to the superconducting coils, and by shrinking the helium inventory down to a sealed, tiny charge—more like a shot glass rather than a swimming pool.

Operationally, this means no routine helium deliveries, no boil-off losses, and no panicked calls when helium prices spike again; your main running costs are electricity and normal maintenance, not slowly boiling money into the atmosphere.

So now it is time to introduce some short explanations and definitions that are frequently used in the marketing context and subsequently also in this paper.

### Sealed micro-helium MRI magnet (“Helium Free” is often used in the marketing language for that)

An imaging magnet (typically 1.5 Tesla) that contains a micro-helium system (Fig. 1, right), where a tiny helium volume is sealed for the life of the magnet and where the Helium does not boil off and evaporate. With that, it never needs refilling, removing the need for future helium supply and related safety logistics for the hospital. The vendors often use the marketing term “HELIUM FREE” for the new magnets they offer, which is technically not correct but refers to the fact that no future helium fills are necessary.

### Conduction cooling (also often used in connection with “Dry Magnets”: a “Helium-Free MRI requires such a cooling method)

Is the dry cooling method for these sealed magnets, where heat is drawn out through solid metal paths into a refrigerated cold plate, instead of using a large tank of liquid helium to absorb the heat, and where the helium gas in the vessel is subsequently refrigerated again?

### High-temperature superconductor (HTS)

Magnet technology using superconductors that work at higher cryogenic temperatures, allowing efficient dry cooling and do not require liquid helium as a cryogen. The higher temperatures required to reach the superconducting state make it possible to use higher-temperature cryostats and require less extensive cooling systems. Not discussed in this paper.

The systems this article refers to are sealed micro-helium magnets that are now also lighter and more compact. While the weight of 1 L liquid Helium is only 0.125 kg, and with that the net weight saving would only be in the order of a little more than 100 kg, the fact that there is no large pressure vessel needed anymore reduces the weight significantly. This suddenly makes the architect your friend instead of your adversary: fewer structural reinforcements, because the magnet weight is now typically in the order of 2.5 tons versus a previous 5 tons, and with that simpler siting, and there is no longer the need for a quench pipe for a sudden release of helium gas (cost of € 500 to € 1000 per meter plus related building changes) [4–7].

From a hospital-economics perspective, the move from large liquid-helium baths to sealed, micro-helium systems eliminates an entire cost category rather than merely trimming it.

The equipment needed to cool down a few liters of Helium is also smaller and cheaper (both operationally and in investment) than the equipment needed for the older-generation magnets.

Older systems could burn through significant budgets over their lifetimes on helium alone (depending on magnet generation, between 0.1 L/day and 2 L/day), especially if boil-off rates or logistics were suboptimal. Sealed designs decouple the scanner’s viability from the helium market drama and supply interruptions that we have seen in recent years.

Energy consumption can also be optimized because the cold mass is smaller and there’s no massive bath to keep at around 4 K, although you still have to pay for the cryocoolers’ power draw [8, 9].

## MRI helium “Free” volume relevance

We have already learned that Helium-Free is not really Helium-Free, and the vendors obviously agree, as they now focus on highlighting the sealed Helium volume. At the most recent radiology conferences, even with less than 1 L versus the competitors' volumes of 7 or 20 L. Is this relevant, or is it where the physics and the practicalities quietly diverge from intuition?

In a sealed, conduction-cooled magnet, that helium is part of the internal thermodynamic plumbing, and under normal conditions, none of it escapes, and you’re not shelling out money for a refill, whether you have 1 L or ten.

So from the radiology department’s daily viewpoint, those numbers barely matter: the budget line for helium is still effectively zero, the service workflow is the same, and nobody is scheduling a “helium day” anymore. The really important change occurred when we dropped from many hundreds of liters in an open bath to single-digit liters in a sealed can; going from 7 to 0.7 L is a factor of 10 but is not particularly relevant [6, 9].

Where volume still has a subtle impact is in worst-case safety scenarios and engineering margins.

If a catastrophic fault ever did release all the helium from the sealed environment, less than 1 L expanding to gas (equal to around 750 L of gas) is slightly less of an asphyxiation and overpressure concern than 7 L, but both are two orders of magnitude gentler than the old “entire swimming pool flashes to gas” scenario that justified massive quench pipes and detailed vent-routing studies.

Likewise, a smaller helium inventory can make manufacturing, shipping, and regulatory classification a bit easier, and can shave a tiny bit off total system mass, but again, the big win is the step down from hundreds of liters to a few—after that, we are in the realm of engineering finesse rather than night-and-day clinical impact.

From a physics-and-operations standpoint, there is no real meaningful difference [4, 6, 8].

In sealed micro-helium MRI magnets, loss of cooling leads to a slow warm-up, with the field typically remaining for many hours, possibly days, depending on the magnet and cryostat design. A 7 L sealed magnet may have a somewhat longer grace period than a 0.7 L design, but in both cases, the dominant factors are thermal insulation and control strategy rather than the exact small helium volume.

## What should you ask?

The question to ask then is, how often does a power outage happen? Can I protect myself for that (battery or generator backup), and is it worth it, since I typically cannot scan during a real power outage anyway?

What I protect with a backup is that the magnet loses its magnetic field and might subsequently require quite a bit of effort and time to get it back up and running to start scanning patients again. For geographies with regular power outages over an extended period of time (> 6 h at a time), this could be critical and could eventually cause the magnet to quench and lose its superconductivity and magnetic field.

It is therefore relevant what happens after that loss. How long will it take to get the system back up and running, and does it require any special equipment, such as a power supply? How long will it take to be fully operational again?

These are the questions that have a significant economic impact, which you need to ask your preferred MRI supplier and your facility manager to calculate and estimate the total cost of ownership and related operational issues.

A tender document for the procurement process of a new MRI system should include the following sentence and ask for additional information that is not normally provided, but that is essential for the total evaluation:

The MRI system shall preferably employ a sealed micro-helium or cryogen-free magnet design to eliminate routine helium refills, quench-vent piping, and helium-related downtime, thereby reducing lifecycle costs and siting complexity. As part of the provided system documents, you need to:

- specify the energy cost per year of the entire MRI magnet system, including the relevant cooling systems;
- the total maintenance cost for the refrigeration units installed in the MRI magnet per year, and the associated downtime for these service works;
- the time that the MRI magnet subassembly system is able to maintain its magnetic field without cooling; the downtime and associated ramp-up costs in case of a magnet quench;
- and the response time after such a quench event.

## Summary

**Fig. 2:**
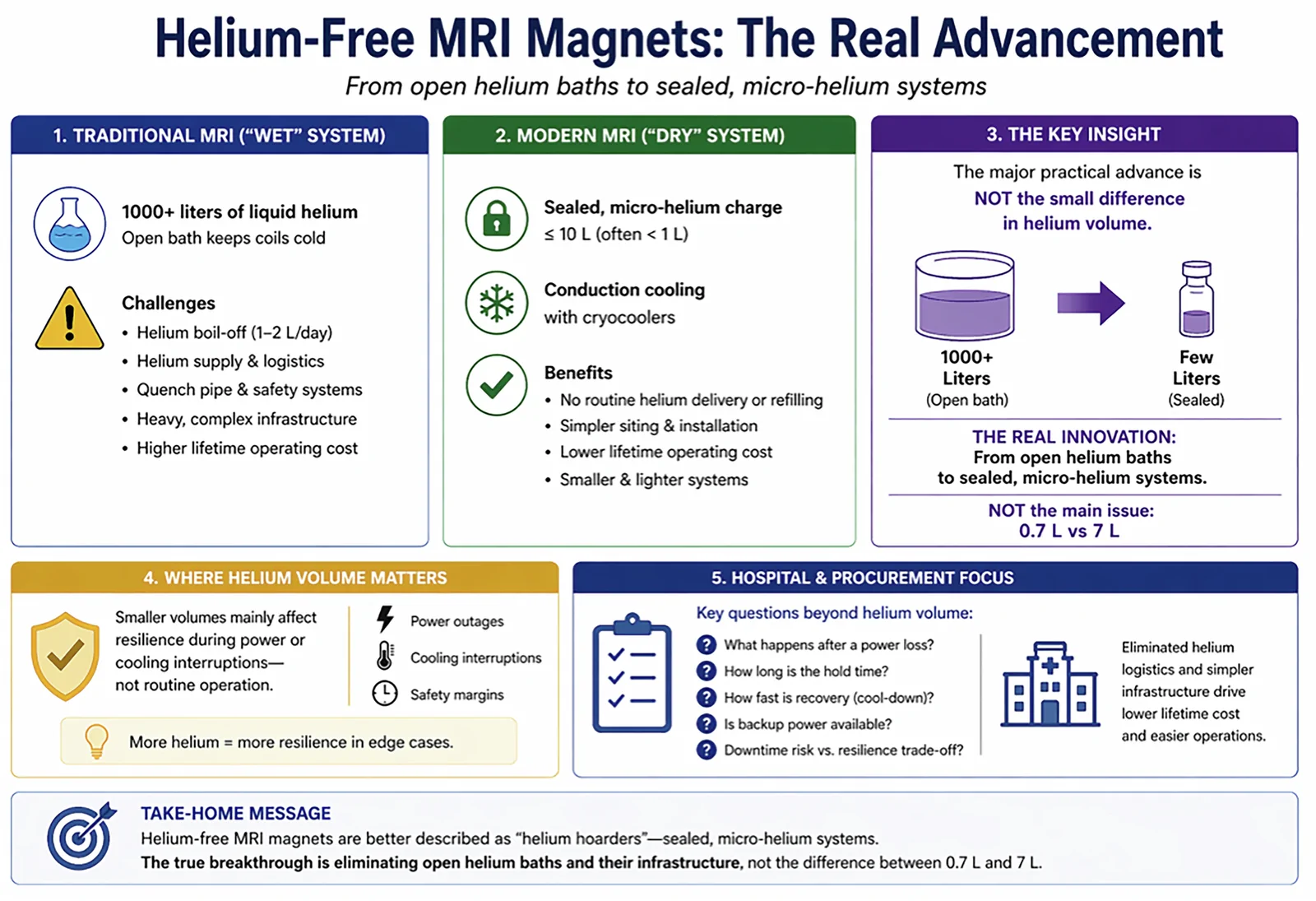
The difference between the traditional “wet” and the new “dry” systems is between 1000 L of liquid helium and just a few liters, leading to handling and operational advantages. “Open” bath in this context means that the magnet generating coils are fully immersed in a liquid helium bath. Both system designs have refrigeration units attached, consuming significant amounts of electricity. There are potential downsides for the dry systems in case of power outages that could lead to cooling interruptions and to the loss of the magnetic field. With that, different questions need to be asked before a decision is made. It could even be economically more reasonable to remain with the traditional “wet” system design

Figure 2 shows the key features of the different magnet designs with respect to the helium volume and cooling of the cryostat, as well as the points where and when the helium volume might matter and which questions a procurement officer should ask.

A classic high-field MRI is a giant superconducting donut living in an ultra-cold helium tub that dominates both building design and a portion of the running costs, while modern dry or micro-helium systems are more like self-contained cryogenic appliances that just happen to weigh a couple of metric tons and can see inside you.

The huge leap in practicality and economics comes from eliminating the large helium bath for the magnet-generating coils and their associated logistics and quench infrastructure, but does not come from fine-tuning the last few liters inside a sealed cryostat.

By the time you’re arguing about this 0.7 versus 7 L, you’ve already joined the post-helium era; you’re just choosing between different sizes of safety margin in a system that, to first order, has stopped bleeding helium [5, 7–9].

Both of these low-volume options avoid the main safety risks of traditional magnets because they do not vent large volumes of helium into the room or through a quench pipe. In a sealed system, the helium is confined inside the magnet; the difference between 1 L and 10 L is tiny compared with legacy 1500–2000 L baths, so oxygen depletion or overpressure risks are essentially eliminated during normal and quench operation. The difference between 0.7 L and 7 L turning to gas is between 500 L and 5000 L of gas over several minutes. In a standard MRI suite with a 60 m^3^ room volume, this is not a significant safety hazard.

Operational resilience mainly depends on the quality of conduction cooling, the cryocooler design, auto-recovery after power loss, and system controls, and not on whether the sealed volume is extremely small or very small.

A slightly larger sealed volume can provide more thermal inertia and a slower warm-up if cooling stops, but modern systems are designed so that automatic recovery and controlled ramp-down make both variants generally robust to power interruptions.

The imaging quality of the MRI system—leaving all other relevant variables unchanged—is neither positively nor negatively affected by the helium volume of the magnet.

And if traditional, older “wet” MRI magnets are significantly cheaper and the siting allows for easy, short quench pipe routing, it could still be economically reasonable and feasible to purchase an “old/wet” magnet. Then you need to ask about additional siting expenses, the difference in the cost for the electricity used for the cooling, and the cost of the Helium that needs to be replenished. This could, for example, be the case when an older magnet type (in place and equipped with a quench pipe) is upgraded with the latest imaging hardware, but the total cost of replacing the magnet is too high.

Readers interested in the physics, more construction details, discussion of the thermal issues, a good presentation of the different coils involved in a shielded and Helium-Free MRI magnet, may find excellent papers in [4, 9–11].

Additionally, recommended articles on magnet differences and related operational aspects are found in [12–14].

## Data Availability

No data is available.
